# The Anti-Amyloidogenic Action of Doxycycline: A Molecular Dynamics Study on the Interaction with Aβ42

**DOI:** 10.3390/ijms20184641

**Published:** 2019-09-19

**Authors:** Alfonso Gautieri, Marten Beeg, Marco Gobbi, Federica Rigoldi, Laura Colombo, Mario Salmona

**Affiliations:** 1 Biomolecular Engineering Lab, Dipartimento di Elettronica, Informazione e Bioingegneria, Politecnico di Milano, Piazza Leonardo da Vinci 32, 20133 Milano, Italy; 2 Department of Molecular Biochemistry and Pharmacology, Istituto di Ricerche Farmacologiche Mario Negri IRCCS, Via Mario Negri 2, 20156 Milano, Italy

**Keywords:** amyloid-beta protein, Alzheimer’s disease, doxycycline, iododoxorubicin, curcumin, molecular dynamics

## Abstract

The pathological aggregation of amyloidogenic proteins is a hallmark of many neurological diseases, including Alzheimer’s disease and prion diseases. We have shown both in vitro and in vivo that doxycycline can inhibit the aggregation of Aβ42 amyloid fibrils and disassemble mature amyloid fibrils. However, the molecular mechanisms of the drug’s anti-amyloidogenic property are not understood. In this study, a series of molecular dynamics simulations were performed to explain the molecular mechanism of the destabilization of Aβ42 fibrils by doxycycline and to compare the action of doxycycline with those of iododoxorubicin (a toxic structural homolog of tetracyclines), curcumin (known to have anti-amyloidogenic activity) and gentamicin (an antibiotic with no experimental evidence of anti-amyloidogenic properties). We found that doxycycline tightly binds the exposed hydrophobic amino acids of the Aβ42 amyloid fibrils, partly leading to destabilization of the fibrillar structure. Clarifying the molecular determinants of doxycycline binding to Aβ42 may help devise further strategies for structure-based drug design for Alzheimer’s disease.

## 1. Introduction

**Figure 1:**
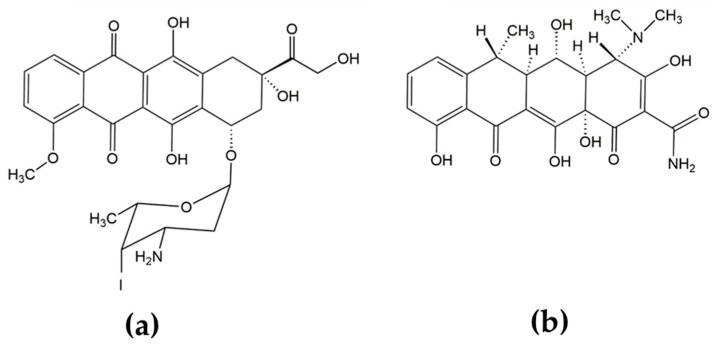
Chemical structures of (**a**) 4′-iodo-4′-deoxydoxorubicin (IDOX) and (**b**) doxycycline.

Interest in the anti-amyloidogenic activity of tetracyclines (TCs) arose from the observation that a structural homolog of doxycycline, the anthracycline anticancer drug 4′-iodo-4′deoxydoxorubicin (IDOX) (Figure 1), produced clinical benefits in patients with immunoglobulin light-chain (LC) amyloidosis. In five patients, a brief course (about a month) of IDOX prevented the progression of amyloidosis and improved their clinical condition over several months [1].

In experimental murine models of amyloidosis in vivo, IDOX specifically accumulated in amyloid deposits of amyloid A, immunoglobulin LC, transthyretin (TTR), amyloid β (Aβ) and β2microglobulin (β2M) but did not bind to the monomeric amyloid precursor [2]. In patients with familial amyloid polyneuropathy (FAP), IDOX co-localized with amyloid deposits and in vitro broke down TTR fibrils from patients into an amorphous precipitate [3]. These data indicated the existence of a universal mechanism of action of IDOX against amyloidosis and made it a potential anti-amyloidogenic drug for all types of amyloidosis [2,4]. However, because of its high toxicity—cardiotoxicity in particular—attempts have been made to identify structurally similar compounds possessing anti-fibrillogenic activity but with a better safety/toxicological profile, already tested in clinical practice.

The resemblance of the polycyclic conjugated structure of TCs with the aglycone moiety of IDOX (Figure 1) prompted initial studies to investigate whether TCs could hinder the pathologic aggregation and propagation of the prion protein [5,6]; these were followed by studies on other amyloidogenic proteins, such as TTR [7,8,9], LC [10], β2M [11,12] and Aβ [13,14,15], which established that: (i) TCs bind to the β-sheet-forming domain of all amyloidogenic proteins, independently of their primary structure; (ii) Inhibition of fibrillogenesis is a result of the immediate interaction of amyloidogenic proteins with the drug, resulting in the formation of stable globular oligomeric species and preventing further fibril growth; (iii) TCs also affect mature fibrils, resulting in their complete or partial disaggregation; (iv) TCs reduce amyloid-induced toxicity in cell culture; (v) Treatment with TCs is often associated with a reduced amyloid load in tissues and provides both improvements in pathological symptoms and prolonged survival in animal models of amyloidoses; (vi) Several preliminary and ongoing clinical trials report improvements in clinical outcomes in patients with different types of amyloidosis in response to TCs [16,17].

TCs have advantages over other newly proposed anti-amyloidogenic drugs, thanks to their well-characterized pharmacological and pharmacokinetic properties and relatively low toxicity. Some TCs efficiently cross the blood–brain barrier [18]. Curiously, this old class of antibiotics was at one point nearly abandoned as a consequence of the development of resistant bacterial strains. Today, however, TCs are involved in more than a hundred clinical trials unrelated to their antimicrobial activity [17]. These include clinical trials focused on the beneficial effects of TC derivatives (particularly doxycycline and minocycline) on amyloidogenic disorders, including Alzheimer (AD), Parkinson (PD), Huntington (HD) diseases and TTR. An increase in the number of publications reporting the successful use of TCs for amyloidoses indicates growing interest in this therapeutic approach. The use of doxycycline as an orphan drug for systemic amyloidosis caused by mutated TTR (Treatment of familial amyloid polyneuropathy. EU/3/12/955) and of β2-microglobulin amyloidosis (Treatment of systemic amyloidosis caused by beta-2 microglobulin. EU/3/12/961) was recently approved by the European Committee for Orphan Medicinal Products [19].

Despite the clear evidence from in vitro experiments that doxycycline can inhibit the aggregation of Aβ42 and disassemble mature amyloid fibrils [15,17], the molecular mechanism responsible for the anti-amyloidogenic effects remains elusive. Here, we report a computational investigation through molecular dynamics (MD) of the possible mechanisms of the anti-amyloidogenic effects of drugs that bind to Aβ fibrils. We compared the results of doxycycline with those of IDOX, curcumin and gentamycin. Curcumin is a well-known molecule that binds to Aβ fibrils [20], inhibits the formation of Aβ oligomers and fibrils and reduces amyloid plaque aggregation in vivo [21,22,23], while gentamycin has no anti-amyloidogenic activity and has served as a negative control in our previous in vitro and in vivo studies [5,24].

## 2. Results and Discussion

### 2.1. Simulation of Aβ Fibrils

**Figure 2:**
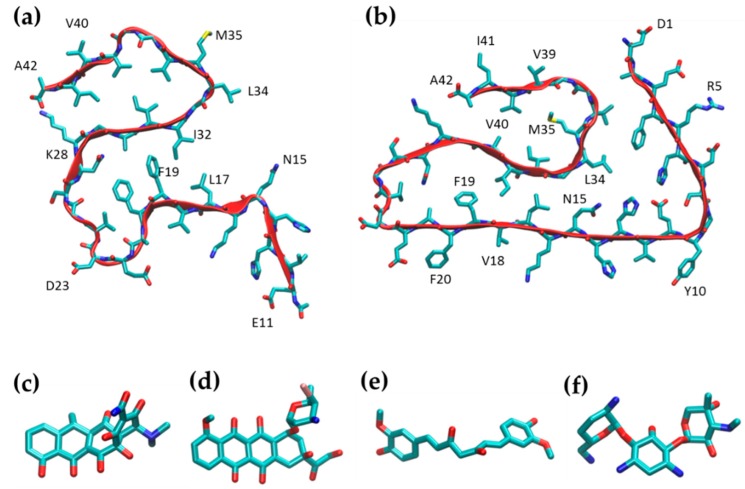
Molecular models of the amyloid Aβ42 fibrils and the small molecules tested. For the simulations, we considered two different polymorphs of Aβ42 fibril structures, with five peptides of the 2MXU PDB entry (**a**) and five peptides of the 5OQV PDB entry (**b**). Structures of candidate anti-amyloidogenic molecules: doxycycline (**c**), IDOX (**d**), curcumin (**e**) and gentamicin (negative control, **f**).

For MD simulations, we started from two different Aβ42 fibril structures (PDB id 2MXU and PDB id 5OQV) obtained from solid-state NMR data and cryo-EM, respectively. The 2MXU structure is made of 12 Aβ peptides and includes residues from Glu11 to Ala42. The NMR structure shows that the fibril core consists of three β-strands (covering residues 12–18, 24–33, and 36–40) arranged in an S-shaped amyloid fold (Figure 2a). This fold generates two hydrophobic cores and has a stabilizing salt bridge between K28 and the C-terminus of A42. The S-shaped 2MXU conformation [25] is distinct from the β-loop-β motif previously suggested for Aβ fibrils. This difference is possibly due to different folding of Aβ42 and Aβ40. In our MD simulations, we considered only the five central peptides of the 2MXU structure.

The S-shaped structure of Aβ42 fibrils has been further confirmed in more recent NMR studies [26] and in the 5OQV structure obtained with cryo-electron microscopy [27]. The 5OQV structure presents two filaments of five peptides each, containing the full sequence of amino acids. The fibril core consists of four β-strands (covering residues 3–22, 26–33, and 36–40,) arranged in an S-shaped amyloid fold (Figure 2b). This fold generates two hydrophobic cores, with a stabilizing salt bridge between K28 and A42, as well as stabilizing polar interactions between H6, E11 andH13. The main differences between the two polymorphs are in (i) the specific amino acids turned towards the core of the fibrils and (ii) the fold of the *N*-terminal portion of the peptides, which in 2MXU points outwards, while the *N*-terminal section of the peptides in the 5OQV structure folds towards the C-terminal region, closing the hydrophobic cavity.

In addition to the MD simulation of the Aβ42 fibrils (control), we ran MD simulations of the Aβ42 fibrils in the presence of high concentrations of candidate anti-amyloidogenic drugs (doxycycline, IDOX and curcumin), with gentamicin as the negative control (Figure 2c-f). All molecules were employed in a 1:1 molar ratio with respect to Aβ peptides.

**Figure 3:**
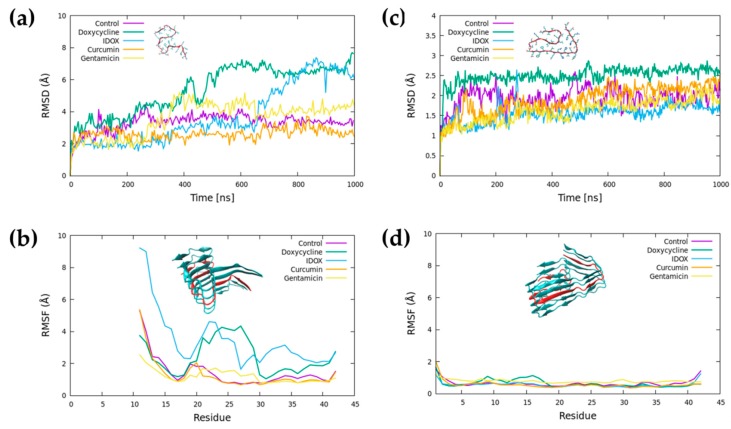
Molecular dynamics (MD) simulations in the absence and presence of the small molecules. Root-mean-square deviation (RMSD) calculated using the C_α_ of each residue (only one trajectory per systems is shown) (**a**,**c**). The root-mean-square fluctuations (RMSFs) (**b**,**d**) were measured using the C_α_ of each residue of the central peptide, excluding the first 200 ns of the MD simulations, and were averaged over triplicate simulations.

Root-mean-square-deviation (RMSD) analysis indicated that both Aβ42 fibrils, in the absence of any small molecule, quickly reached a stable structure throughout the MD simulation (Figure 3a,c). The pattern was similar for the fibril in the presence of curcumin and gentamicin. Conversely, doxycycline and IDOX led to partial destabilization of fibril structure of the 2MXU polymorph within the simulated time frame.

The root-mean-square fluctuation (RMSF) indicates the per-residue atomic fluctuation over the average position (Figure 3b,d). For the 2MXU structure, the *N*-terminal segment of the Aβ42 peptide (residues 11–15) showed more significant fluctuations than the central segment of the peptide, in contrast with what observed for the 5OQV structure. This was due to the higher solvent exposure and the lack of stabilizing interactions. Conversely, the *C*-terminus showed low structural fluctuations thanks to the stable salt bridge between the side chain of K28 and the carboxyl group of A42. The presence of curcumin and gentamicin did not alter the RMSF profile for the control system.

The presence of doxycycline and IDOX led to a significant increase in the average structural fluctuation of the central region. Doxycycline, in particular, destabilized the hydrophobic core covering residues N15–A30, while the destabilizing effect of IDOX was seen through the whole peptide. None of the molecules altered the stability of the 5OQV polymorph during the simulation.

**Figure 4:**
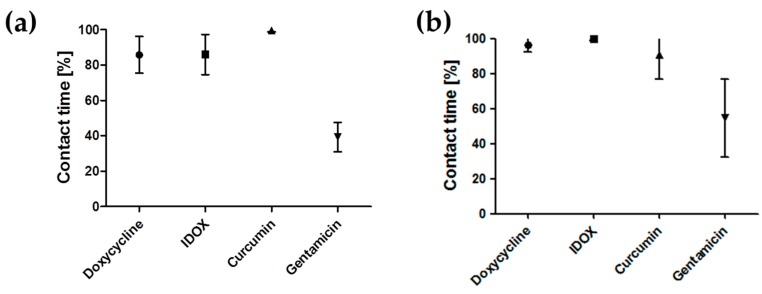
Average contact time between anti-amyloidogenic molecules and the two different polymorphs of Aβ42 fibrils, 2MXU (**a**) and 5OQV (**b**) during the MD simulation. The contact time is the amount of time (as a percentage) during which the molecule is in contact with the fibril. The 5 ns window is meant to exclude sporadic contacts due to the high simulated concentration.

To provide the first indication of drug binding to the Aβ42 fibrils, we quantified the contact time between the small molecules and the fibrils. In our definition, contact is achieved when the molecule is within 3.5 Å from the peptides for at least 5 ns, excluding the first 200 ns of MD simulation from the analysis (see Figure 4a,b). This clearly shows that doxycycline, IDOX and curcumin remained in stable contact with both fibril polymorphs throughout the simulations. Gentamicin, on the other hand, formed sporadic, non-localized contacts with the Aβ42 fibrils.

### 2.2. Doxycycline Binding

**Figure 5:**
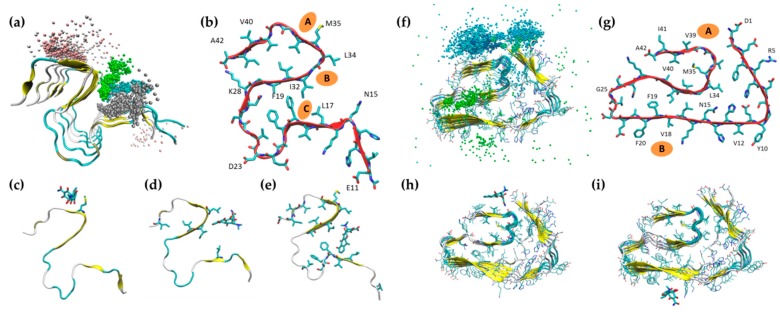
Doxycycline binding sites on Aβ42 fibrils. Superposition of doxycycline coordinates (center of mass) during the MD simulations (excluding the first 200 ns) in the presence of the two polymorphs of Aβ42 fibrils (2MXU, (**a**); 5OQV, (**f**).) Panels (**b)** and (**g)** show the main interaction sites. When simulated with the 2MXU structure, doxycycline stably bound to the side chain of M35 (**c**), within the groove formed by I32 and L34 (**d**) and within the groove formed by F19 and L17 (**e**). In the simulations with the 5OQV structure, doxycycline stably interacted with the hydrophobic groove formed by V39 and I41 (**h**), as well as with the groove formed by F20 and V18 (**i**).

To examine the binding of doxycycline to Aβ42 fibrils, we superposed the position of doxycycline molecules throughout the MD simulation (Figure 5a for 2MXU and 5f for 5OQV). On the basis of the formation of ligand clusters close to the fibrils during the simulation, we identified three main binding sites where the molecules formed highly stable interactions with the 2MXU fibril throughout the MD simulations (Figure 5b).

A first binding position was on the outer face of the smaller hydrophobic pocket, near the aligned side chains of M35 residues (Figure 5c). This position was stabilized by hydrogen bonds between the oxygen-rich lower peripheral region of doxycycline and the backbone of the Aβ fibril, as well as by hydrophobic contacts between the doxycycline carbocyclic rings and the side chains of methionine residues.

A second binding region was at the solvent-exposed face of the larger hydrophobic core of the fibril (defined by residues from N15 to L34). In this region, a first stable binding site was determined by the hydrophobic groove between I32 and L34 (Figure 5d). This position was stabilized by hydrophobic contacts between the D ring of doxycycline and the side chains of I32 and L34. Partial stabilization was also provided by hydrogen bonds between the doxycycline lower periphery and the fibril backbone.

The solvent-accessible side of the hydrophobic core hosted a second binding site defined by the hydrophobic groove between L17 and F19 residues (Figure 5e) and stabilized by hydrophobic interactions between the D-ring of doxycycline and the side chains of the L17 and F19.

When doxycycline molecules were present during the 5OQV fibril simulation, there was a stable interaction of the molecule with two regions, both characterized by solvent-exposed hydrophobic amino acids (Figure 5f,g). The first area was near the *N*-terminal, where doxycycline formed polar interactions with Glu1, while its hydrophobic rings interacted with residues Val39 and Ile41 (Figure 5h). The second area was bounded by Phe20, Val18 and Lys16 (Figure 5i).

We estimated 2MXU–doxycycline binding energy using the Molecular Mechanics Poisson-Boltzmann Surface Area MM-PBSA approach (Table 1). The binding energy was similar for the three binding sites identified. In all cases, the stabilizing electrostatic term (ΔG_EL_) was counterbalanced by an unfavorable electrostatic contribution to the solvation-free energy (ΔG_Sol-P_). In all these cases, therefore, the main stabilizing contribution to doxycycline binding was due to non-polar interactions (ΔG_vdW_). The binding energy for doxycycline was comparable to those previously obtained for curcumin (from −4 to −16 kcal/mol) [28] and for the anti-amyloidogenic compound wgx-50 (from −12 to −35 kcal/mol) [29]. In the doxycycline interaction with 5OQV fibrils, despite the long interactions between the molecules and the fibrils, we could not identify any specific stable conformation of doxycycline, suggesting an unspecific interaction. Therefore, no binding energies were calculated.

### 2.3. Iododoxorubicin Binding

**Figure 6:**
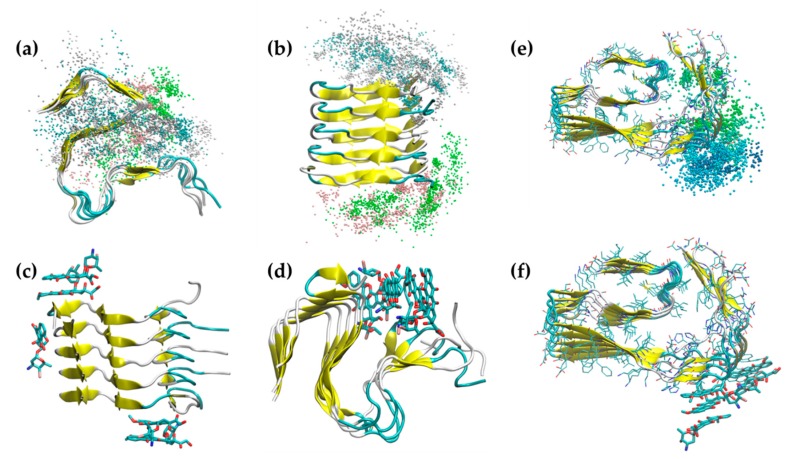
Iododoxorubicin binding to (**a**–**d**) 2MXU and (**e**,**f**) 5OQV fibril structures. Superposition of iododoxorubicin coordinates (center of mass) during the 2MXU MD simulations (**a**,**b**) and snapshots of the main binding regions (**c**,**d**). Superposition of IDOX coordinates during the 5OQV MD simulation (**d**) and a snapshot of the main binding area (**f**).

The superposition of IDOX coordinates during the MD simulation (Figure 6a,b,e) indicated that the molecules kept tight contact throughout the simulations with both polymorphs of the Aβ fibril. In the simulations with the 2MXU fibrils, we observed three major binding regions. IDOX molecules formed stable interactions with the top and bottom faces of the fibrils in correspondence with the two hydrophobic pockets, one defined by the residues from K28 to A42, the other by the residues from L17 to I32 (Figure 6c). These contacts were stabilized by the hydrophobic interactions between the rings of IDOX and the side chains of the Aβ fibrils. A third binding region corresponded to the solvent-exposed face of the larger hydrophobic core (Figure 6d). Differently from doxycycline, IDOX molecules quickly packed together in a cluster, forming stable π–π interactions between the aromatic rings of different IDOX molecules, leading to quick self-aggregation. The binding energy estimated with MM-PBSA (Table 2) showed tighter binding than with doxycycline and similar binding energy for the three spots, dominated by non-polar interactions (ΔG_vdW_). The lower binding energy for IDOX than for doxycycline suggests stronger binding, consistent with the observation that IDOX showed anti-amyloidogenic properties at nanomolar concentrations [2], while micromolar concentrations were needed to obtain the same effects with doxycycline [15]. When IDOX molecules were simulated in the presence of 5OQV fibrils, there was a highly stable interaction in the region from Gly10 to His14, with significant interaction involving the aromatic ring of Tyr10 and the hydrophobic side chain of Val12 (Figure 6f). Similarly to the simulations with 2MXU fibrils, IDOX molecules quickly packed together in a cluster, forming stable π–π interactions between the aromatic rings, also in the presence of 5OQV fibrils. However, with this polymorph, IDOX showed no interactions in specific positions, with a well-defined pose.

### 2.4. Curcumin Binding

**Figure 7:**
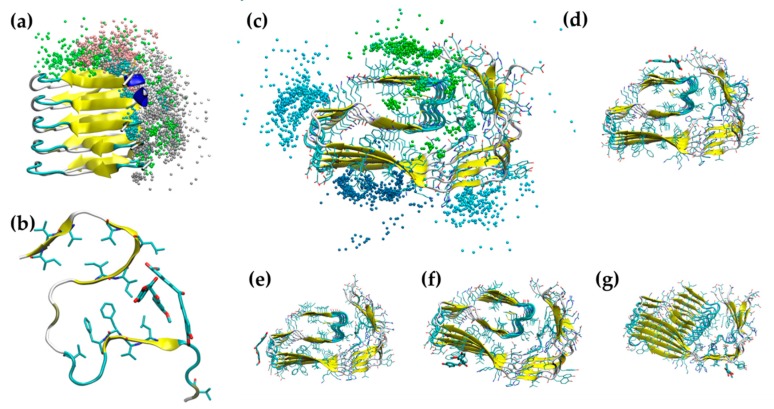
Curcumin binding to (**a,b**) 2MXU and (**c**–**g**) 5OQV Aβ fibrils. Superposition of curcumin coordinates (center of mass) during the 2MXU MD simulations (**a**) and a snapshot of the main binding area (**b**). Superposition of curcumin coordinates during the 5OQV MD simulation (**c**) and snapshots of the main binding area (**d**–**g**).

Curcumin molecules formed continuous contacts with Aβ fibrils during the MD simulations (Figure 7a,c). In the case of the 2MXU polymorph, the main region of interaction was at the solvent-accessible face of the main hydrophobic core, where the molecules formed a tight cluster (Figure 7b). A second interaction region was in the upper face of the fibril in correspondence with the two hydrophobic pockets (Figure 7a). In the simulations with the 5OQV polymorph, four different interactions sites were identified, corresponding to residues V39–I41 (Figure 7d), the kink near residue G25 (Figure 7e), the hydrophobic groove bounded by V18–F20 (Figure 7f) and the hydrophobic groove bounded by Y10 and V12 (Figure 7g). Although curcumin molecules maintained tight contact with both polymorphs of amyloid fibrils, no stable localized binding sites were observed. These results suggest that curcumin strongly bound the exposed hydrophobic residues of the fibrils, although in an unspecific way. We observe that curcumin quickly “decorated” the surface of the fibril, thus possibly preventing further aggregation of the peptides into fibrils.

## 3. Materials and Methods

### 3.1. Molecular Models

The two structural models of Aβ fibrils in this study were generated starting from two experimental structures of the Aβ42 amyloid fibril (Protein Data Bank identification codes 2MXU [30] and 5OQV [27]) (Figure 2a). The 2MXU PBD structure consists of a single filament of 12 peptides, each lacking the first 10 amino acids. The current study considered only five peptides in the simulations, to reduce computational costs. The 5OQV structure represents a different polymorph of Aβ42 and consists of a double filament of five peptides each, all including the full sequence. For this structure too, a single filament of five peptides was considered for the present study.

The proteins were modeled using the Amber ff14SB force field [31], while the force field parameters for doxycycline (in the zwitterionic form), IDOX, curcumin and gentamicin (Figure 2c–f) were generated using the general amber force field (GAFF) [32].

### 3.2. Molecular Dynamics

The complexes of Aβ fibrils modeled with the different ligands were built by introducing five ligand molecules to achieve a 1:1 molar ratio, as in previous in vitro studies [15]. The initial position of the ligands was chosen randomly 10 Å away from the Aβ fibril (see Supplementary Information).

In addition to the eight molecular systems (two Aβ fibrils complexed with the four ligands), we also modeled the Aβ fibril without any ligands as a control system. All molecular models were solvated with TIP3P water molecules, and the system was neutralized with the addition of 10 Na^+^ ions. This resulted in systems of ≈60,000 atoms in a simulation box with initial dimensions approximately 80 × 80 × 80 Å^3^. All systems were generated and simulated in triplicate (see Figure S1).

The systems were modeled following protocols described in previous studies [33,34,35,36,37]. They were all minimized and equilibrated for 100 ps using the NAMD code [38] under constant pressure and temperature (NPT) to relax the volume of the periodic box. In the NPT simulations, the temperature was controlled at 300 K using a Langevin dynamics thermostat, and the pressure was controlled at 1 bar using a Nose–Hoover–Langevin barostat. MD simulations were run using a 2 fs time step, a 12 Å non-bonded cut-off, rigid bonds and particle-mesh Ewald long-range electrostatics. During minimization and NPT equilibration, the C_α_ atoms of the protein were restrained by a 10 kcal·mol^−1^ Å^−2^ spring constant to prevent protein diffusion. Also, one atom for each ligand was restrained to the initial position to avoid the ligand wandering in the equilibration phase. Subsequently, for the production run, we used ACEMD [39] on an NVIDIA Kepler K40 GPU for a total time of 1 µs. A longer time step of 4 fs was used with the hydrogen mass repartitioning scheme implemented in ACEMD. All other parameters (temperature, non-bonded cut-off and PME) were kept the same as in the equilibration phase.

The stability and evolution of the systems were assessed by monitoring the RMSD of the Aβ fibrils. The effects of the ligands on the stability of the Aβ fibrils were also examined by calculating the RMSF of the amino acids belonging to the central peptide, excluding the first 200 ns. The contacts between the Aβ fibrils and the ligands were calculated on a geometrical basis (less than 3.5 Å distance between any heavy atoms of the molecule and the peptide, and contact maintained for at least 5 ns). For the analysis, we used in-house tcl-scripts [40,41] in VMD [42]. The MM-PBSA single trajectory approach implemented as a script (MMPBSA.py) [43] in AmberTools18 [44] was used to calculate the binding energy.

## 4. Conclusions

Using MD simulations, we investigated the binding of three small molecules to two different structures of the Aβ fibril, their effect on the fibril stability and the preferred binding sites. Except for gentamicin, the small molecules all stably bound to the amyloid fibrils. Gentamicin formed only sporadic contacts with the amyloid fibrils, in agreement with previous observations from our laboratory [6,24] that this small molecule does not affect Aβ fibril stability. Doxycycline bound tightly to the Aβ fibrils, leading to partial destabilization of the fibril structure, particularly in the hydrophobic core covering residues N15–A30. This is in good agreement with previous reports that TCs—particularly doxycycline—can inhibit the aggregation of Aβ42 and disassemble mature amyloid fibrils [15,17,45]. Thanks to the stability of doxycycline binding to the 2MXU polymorph, we were able to identify three main binding sites: one corresponding to the side chains of methionine 35, and two on the solvent-accessible side of the major hydrophobic core of the Aβ fibril. Interestingly, very similar binding sites have been described for an unrelated compound (wgx-50 extracted from *Sichuan pepper*) binding to Aβ fibrils in the hairpin conformation [29], confirming the importance of these sites. These sites are hidden in the 5OQV fibril, so doxycycline binds the exposed hydrophobic amino acids unspecifically. IDOX and curcumin, two other anti-amyloidogenic compounds, stably bound to both polymorphs, and IDOX led to partial destabilization of the 2MXU fibril structure.

All the anti-amyloidogenic compounds studied interacted strongly with both fibrillar structures, “decorating” their surface in correspondence with the exposed hydrophobic residues. Binding to the fibril surface could hinder surface-catalyzed formation of oligomers [46]. IDOX and curcumin could also hinder fibril elongation, since they bound to the fibril ends as well. Doxycycline and IDOX showed potential for destabilization of the fibrils, although only for the 2MXU polymorphs. This might be due to the tighter packing of the 5OQV structure which, in the absence of any compounds, was more stable than the 2MXU structure.

The molecular simulations described here confirm the molecular basis of experimental observations of the anti-amyloidogenic activity of doxycycline. However, its pharmacological efficacy may also rely on broader multitarget activity, including inflammation, reactive oxygen species (ROS) generation, apoptosis, uncoupling of metal homeostasis and possibly an indirect effect on amyloid fibrils [7,18].

## Figures and Tables

**Table 1:** Doxycycline–Aβ fibrils (2MXU) binding energy from MM-PBSA. ΔG_EL_ represents the electrostatic interaction energy, ΔG_vdW_ the van der Waals interaction, ΔG_Sol-P_ the polar contribution to the solvation energy, ΔG_Sol-NP_ the non-polar contribution to the solvation energy, and ΔG_bind_ the total binding energy. All values are in kcal/mol.

Binding Site	ΔG_vdW_	ΔG_EL_	ΔG_Sol-P_	ΔG_Sol-NP_	ΔG_bind_
M35	−39.4 ± 3.7	−19.7 ± 5.9	46.9 ± 6.7	−3.3 ± 0.2	−15.5 ± 3.4
I32–L34	−35.2 ± 2.5	−17.3 ± 4.8	38.7 ± 4.8	−3.2 ± 0.1	−16.8 ± 3.3
F19–L17	−44.9 ± 9.8	−15.7 ± 9.4	40.7 ± 5.5	−3.8 ± 0.4	−23.7 ± 5.0

**Table 2:** Iododoxorubicin–Aβ fibrils (2MXU) binding energy from MM-PBSA. ΔG_EL_ indicates the electrostatic interaction energy, ΔG_vdW_ the van der Waals interaction, ΔG_Sol-P_ the polar contribution to the solvation energy, ΔG_Sol-NP_ the non-polar contribution to the solvation energy, and ΔG_bind_ the total binding energy. All values are in kcal/mol.

Binding Region	ΔG_vdW_	ΔG_EL_	ΔG_Sol-P_	ΔG_Sol-NP_	ΔG_bind_
K28–A42	−65.6 ± 5.5	−12.6 ± 7.8	45.4 ± 7.4	−5.1 ± 0.2	−38.0 ± 5.3
L17–I32	−60.6 ± 5.7	−18.1 ± 11.3	47.9 ± 10.3	−4.9 ± 0.4	−35.9 ± 4.3
Hydrophobic pocket	−52.4 ± 5.4	−10.8 ± 4.6	36.0 ± 5.2	−4.2 ± 0.4	−31.6 ± 4.4

## Supplementary Materials

Supplementary materials can be found at https://www.mdpi.com/1422-0067/20/18/4641/s1.

## Abbreviations

IDOX	4′-do-4′deoxydoxorubicin
TCs	tetracyclines
