# Supplementary material for: The Anti-Amyloidogenic Action of Doxycycline: A Molecular Dynamics Study on the Interaction with Aβ42
Source: Int J Mol Sci. 2019 Sep 19;20(18):4641. doi: 10.3390/ijms20184641 (PMC6769662; doi:10.3390/ijms20184641)
Supplement: Supplementary file 1 [file ijms-20-04641-s001.pdf]

Supplementary Information

The anti-amyloidogenic action of Doxycycline: a molecular dynamics study on the interaction with Abeta

Alfonso Gautieri[a,*], Marco Gobbi[b], Marten Beeg[b], Federica Rigoldi[a], Laura Colombo[b], Mario Salmona[b,*]

[a] Biomolecular Engineering Lab, Dipartimento di Elettronica, Informazione e Bioingegneria, Politecnico di Milano, Piazza Leonardo da Vinci 32, 20133 Milano (Italy)

[b] Istituto di Ricerche Farmacologiche Mario Negri IRCCS, Via Mario Negri 2, 20156 Milano (Italy)

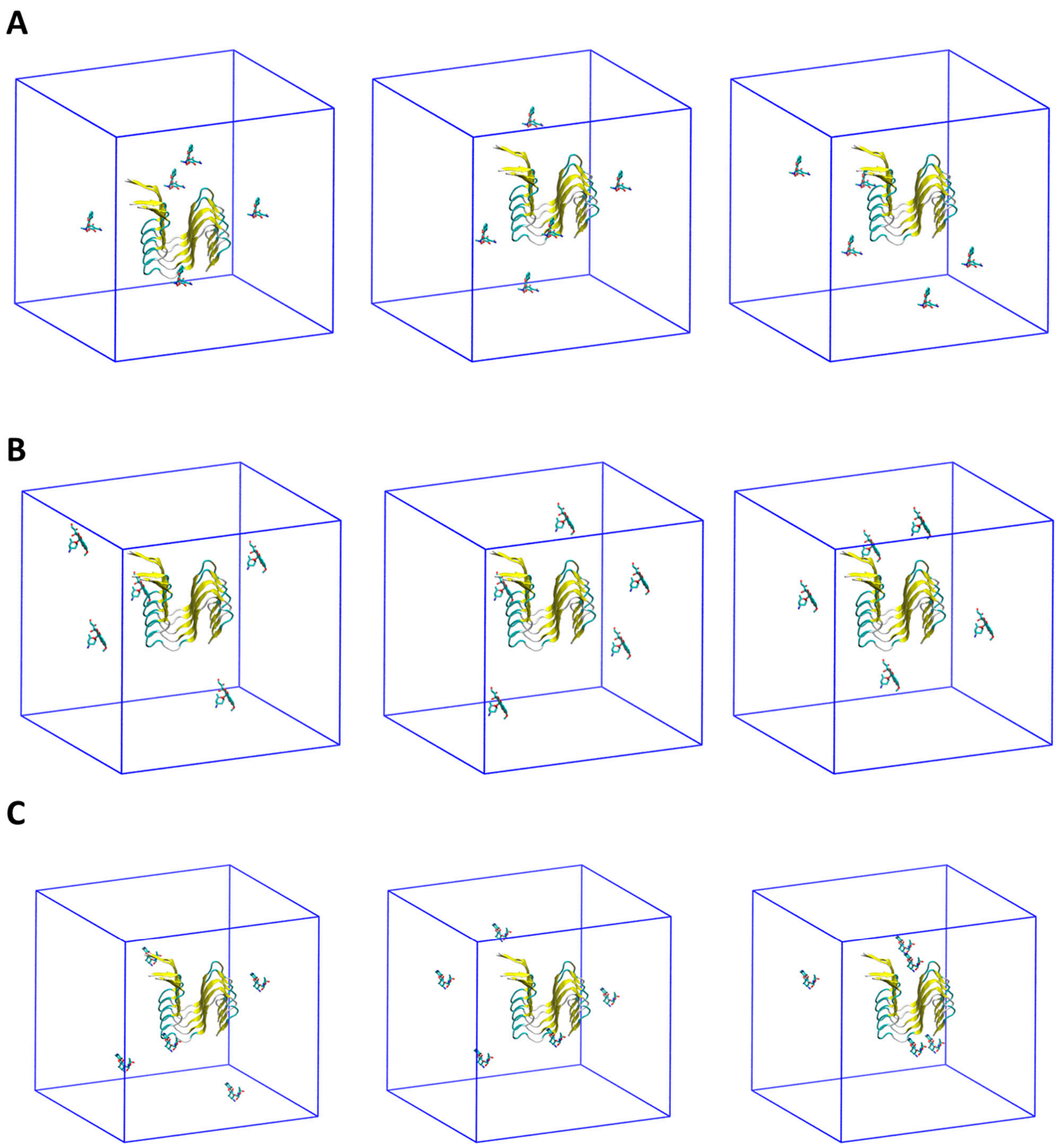

Figure S1. Starting configurations for the molecular dynamics simulation of Aβ42 in the presence of doxycycline (A), IDOX (B) and gentamicin (C).

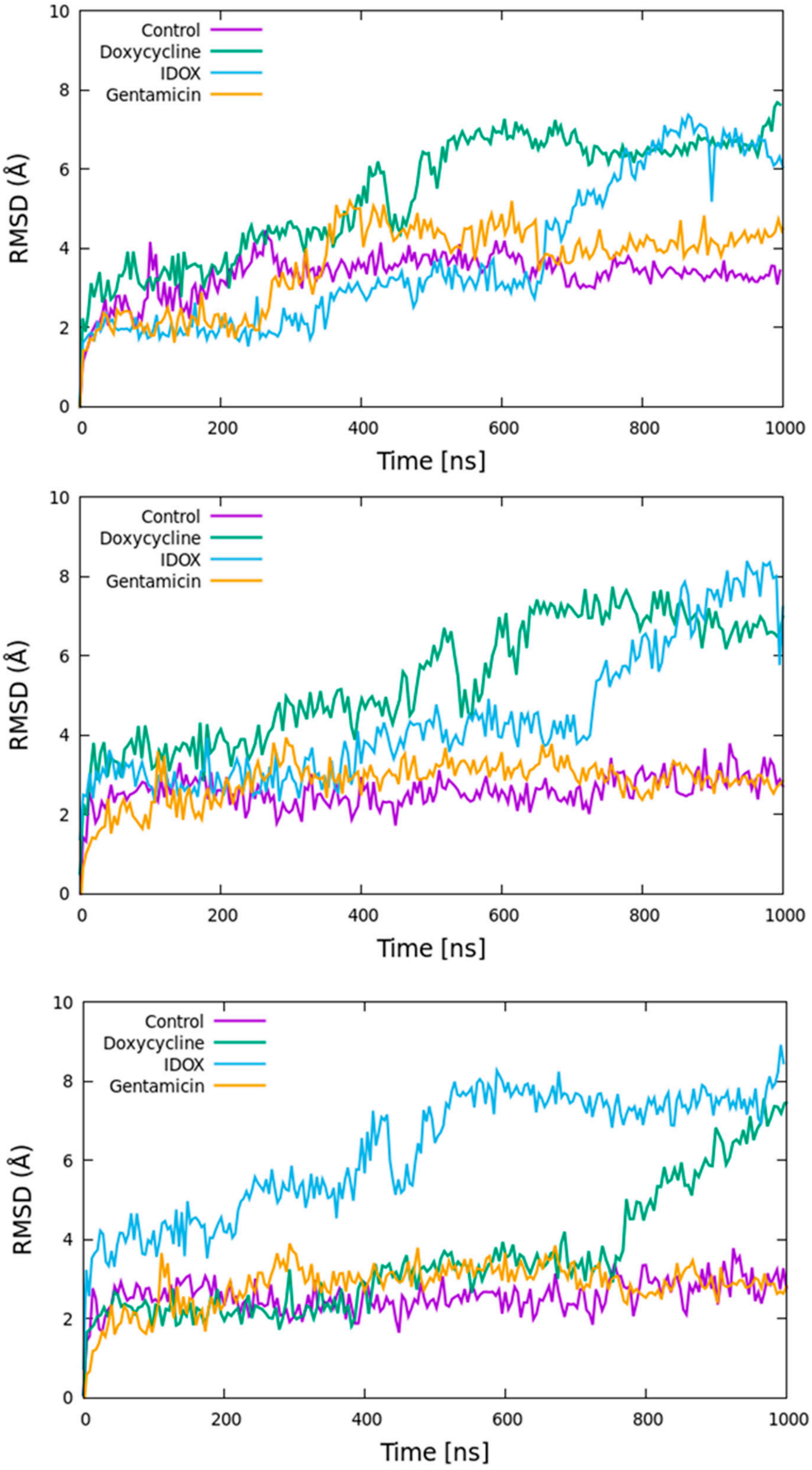

Figure S2. RMSD calculated for the three replicas of each system

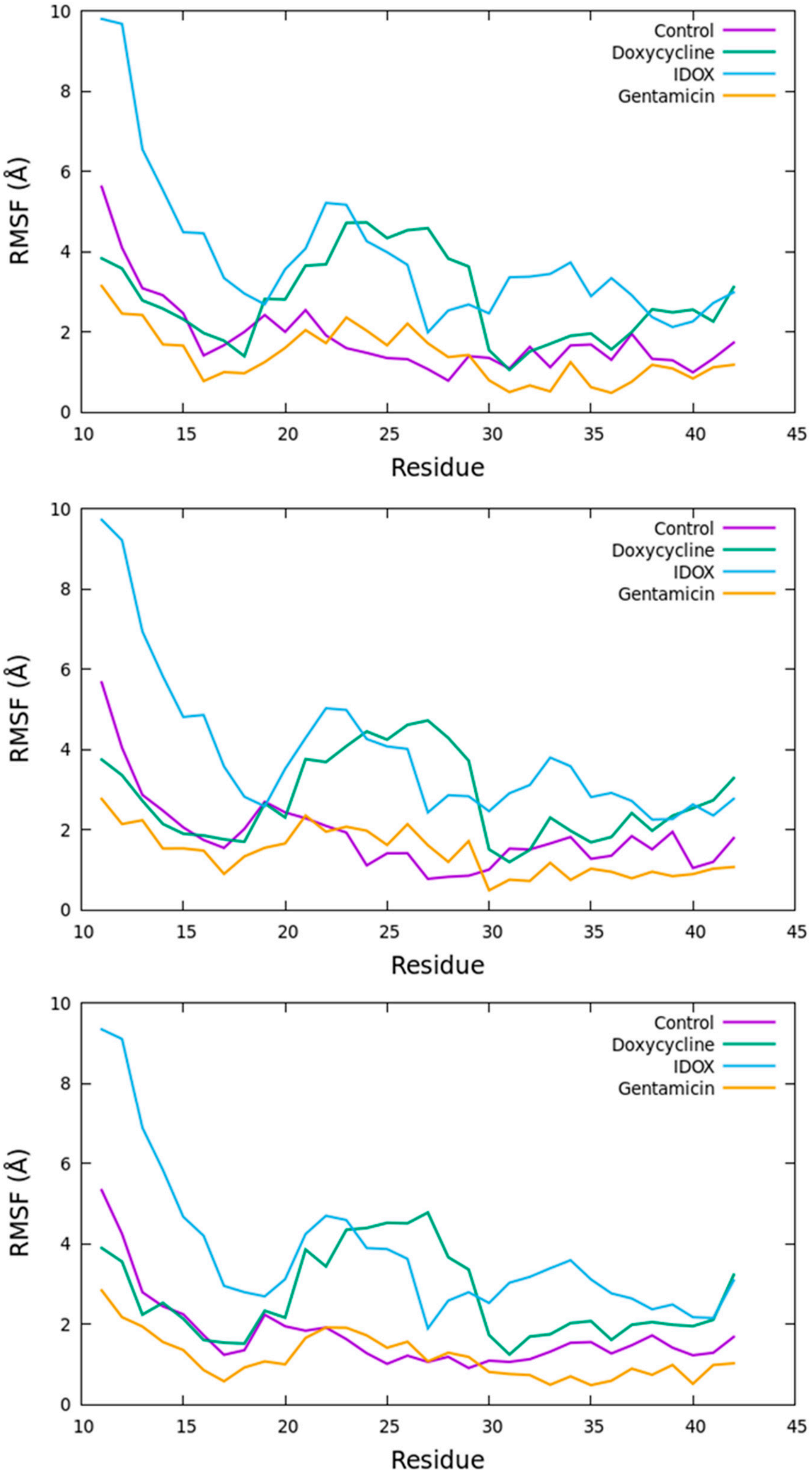

Figure S2. RMSF calculated for the three replicas of each system.
